# São Tomé and Príncipe on the Verge of Eliminating Lymphatic Filariasis as a Public Health Problem: Evidence from IDA Impact Assessment Surveys

**DOI:** 10.3390/tropicalmed11100262

**Published:** 2026-09-23

**Authors:** Didier Bakajika, Alzíra Rosário, Jessica Vicente Fernandes, Lamine Diawara, Jean-Paul Tambwe Mangala, Jean Kale Kalenga, Vilfrido Santana Gil, Dyesse Yumba, Honorat Zouré, Elizabeth Juma, Abdoulaye Diarra, Jonathan King, Jorge Cano

**Affiliations:** 1 Expanded Special Project for Elimination of Neglected Tropical Diseases (ESPEN), World Health Organization, Regional Office for Africa, Brazzaville P.O. Box 06, Democratic Republic of the Congo; diawarala@who.int (L.D.); yumbad@who.int (D.Y.); zoureh@who.int (H.Z.); jumae@who.int (E.J.); canoj@who.int (J.C.); 2 National Programme for the Control of Neglected Tropical Diseases, Ministry of Health, Sao Tome P.O. Box 23, Sao Tome and Principe; alzirarosario@hotmail.com (A.R.); jessicavicentefernandes@gmail.com (J.V.F.); 3 Preventive Chemotherapy Neglected Tropical Diseases Programme, Ministry of Public Health, Hygiene, and Social Welfare, Kinshasa P.O. Box 8081, Democratic Republic of the Congo; jptambwe@yahoo.fr (J.-P.T.M.); jeankale2003c@gmail.com (J.K.K.); 4 São Tomé and Príncipe Country Office, World Health Organization, Sao Tome P.O. Box 287, Sao Tome and Principe; santanagilv@who.int (V.S.G.); diarraa@who.int (A.D.); 5 Department of Malaria and Neglected Tropical Diseases, World Health Organization, CH-1211 Geneva, Switzerland; kingj@who.int

**Keywords:** lymphatic filariasis, elimination, mass drug administration, IDA regimen, surveillance, impact assessment, São Tomé and Príncipe

## Abstract

Accelerated efforts to eliminate lymphatic filariasis (LF) as a public health problem have been supported by the introduction of the triple-drug regimen of ivermectin, diethylcarbamazine and albendazole (IDA) in endemic settings. In São Tomé and Príncipe, nationwide mass drug administration (MDA) with diethylcarbamazine and albendazole was implemented in 2018, followed by IDA in 2019 and 2020. This study assesses progress towards elimination using post-MDA impact assessment surveys conducted after cessation of treatment. Cross-sectional surveys were conducted among adults aged 20 years and older in 2022 and again between December 2024 and January 2025. Circulating filarial antigen (CFA) was detected using the filarial test strip (FTS). Individuals who tested positive were examined for microfilaremia using nocturnal calibrated thick blood smear microscopy. MDA coverage data were obtained from national programme records, and morbidity was assessed by clinical screening during the surveys. Three rounds of nationwide MDA achieved high epidemiological coverage (86.4% in 2018, 74.2% in 2019 and 80.0% in 2020). The impact assessment surveys conducted in 2022 evaluated 14,132 adults, with 21 individuals (0.15%) testing positive for CFA, while the follow-up survey conducted between December 2024 and January 2025 assessed 14,653 adults and detected seven positive cases (0.05%). No microfilariae were detected among the 28 antigen-positive individuals examined using nocturnal calibrated thick blood smears. Clinical screening during the surveys identified 190 cases of lymphoedema and nine cases of hydrocoele. Infection indicators remain well below WHO decision thresholds, suggesting that LF transmission is unlikely to be sustained. São Tomé and Príncipe appears to be close to eliminating LF as a public health problem. However, strengthening morbidity management services will be essential to support the preparation of the national elimination dossier.

## 1. Introduction

Lymphatic filariasis (LF) is a mosquito-borne neglected tropical disease primarily caused by the filarial nematode *Wuchereria bancrofti* [1]. Chronic infection can lead to lymphoedema of the limbs and hydrocoele in adult males, both of which cause long-term disability, social stigma and economic hardship within affected communities [2]. Lymphatic filariasis has historically affected hundreds of millions of people living in tropical and subtropical regions [3]. To address this burden, WHO established the Global Programme to Eliminate Lymphatic Filariasis (GPELF) in 2000 with the aim of eliminating the disease as a public health problem by interrupting transmission and providing morbidity management and disability prevention (MMDP) services for affected individuals [4,5,6].

The main strategy for interrupting transmission is annual MDA targeting at-risk populations [7]. Traditionally, MDA involved a two-drug regimen of albendazole combined with either ivermectin or diethylcarbamazine, depending on the epidemiological context. WHO has recently recommended the triple-drug regimen consisting of ivermectin, diethylcarbamazine, and albendazole (IDA) for use in settings where onchocerciasis and loiasis are absent [8]. Clinical and field studies have demonstrated that the IDA regimen eliminates microfilariae more rapidly than two-drug regimens and can accelerate progress towards elimination when high treatment coverage is achieved [9,10,11,12,13].

Monitoring and evaluation are essential components of the global elimination strategy because they provide evidence to inform programmatic decision-making and document progress towards elimination [4,6]. WHO recommends a series of epidemiological assessments following MDA to determine whether infection levels have declined to thresholds below which transmission is unlikely to persist [14]. In settings where the IDA regimen has been implemented, impact assessment surveys can provide valuable evidence on reductions in infection prevalence after treatment campaigns [14,15].

São Tomé and Príncipe, an island nation in the Gulf of Guinea with a relatively small and geographically contained population, has historically reported evidence of LF transmission caused by *Wuchereria bancrofti*. Baseline mapping surveys conducted in 2015 using BinaxNOW Filariasis immunochromatographic test (ICT; Abbott Diagnostics Scarborough, Inc., Scarborough, ME, USA identified positive cases across multiple districts and communities on both São Tomé and Príncipe islands, with 1413 individuals tested across 14 localities in all seven districts and 23 antigen-positive individuals detected, with at least one positive result in every district, confirming the endemic status of these districts prior to the initiation of MDA. Following WHO recommendations, a district was classified as endemic when at least one surveyed community recorded at least one antigen-positive result; on this basis, all seven districts were classified as endemic and were consequently targeted for MDA. The per-locality mapping results are presented in Table S1, and the pre-MDA endemicity status is shown in Figure S1. In line with global elimination strategies, the Ministry of Health (MoH) implemented three consecutive rounds of nationwide MDA between 2018 and 2020 [16,17,18]. The first round in 2018 used the two-drug regimen of diethylcarbamazine and albendazole, followed by two rounds of the triple-drug (IDA) regimen in 2019 and 2020. The interventions were designed to quickly reduce the parasite reservoir in the human population and interrupt transmission.

To evaluate the impact of these interventions and guide programmatic decision-making, an IDA impact assessment survey was conducted in 2022 among adults aged 20 years and older using CFA detection. A follow-up survey was subsequently conducted between December 2024 and January 2025 to assess whether infection indicators remained below thresholds consistent with sustained transmission following cessation of MDA. This study describes the epidemiological findings from these assessments and documents the progress made by São Tomé and Príncipe towards validation of LF elimination as a public health problem.

## 2. Materials and Methods

### 2.1. Study Setting

São Tomé and Príncipe is a small island nation in the Gulf of Guinea off the west coast of Central Africa. It consists of two main islands, São Tomé and Príncipe, with an estimated population of 220,000 inhabitants. Administratively, the country is divided into seven health districts: Água-Grande, Mé-Zóchi, Lobata, Cantagalo, Caué, Lembá, and the Autonomous Region of Príncipe. Its tropical climate, characterized by high humidity and rainfall, provides favourable ecological conditions for mosquito vectors such as *Anopheles* species, which are known to drive the transmission of *W. bancrofti* in São Tomé and Príncipe [18].

### 2.2. National Lymphatic Filariasis Elimination Programme and MDA

After evidence of LF transmission was found in the country [19], the MoH implemented a national elimination programme aligned with the WHO GPELF strategy [18]. MDA was conducted nationwide for three consecutive years. The first round in 2018 used a two-drug regimen consisting of diethylcarbamazine and albendazole. Subsequent rounds in 2019 and 2020 used the IDA triple-drug regimen in line with WHO recommendations aimed at accelerating interruption of transmission [20]. Treatment was delivered through community-based distribution targeting eligible populations across all health districts in the country. In line with WHO guidance, the three medicines were administered at standard single doses of diethylcarbamazine (6 mg/kg), albendazole (400 mg) and, for the IDA rounds, ivermectin (200 µg/kg, dosed by height using a dose pole); pregnant women, children below the nationally applied age or height threshold and severely ill individuals were excluded [20].

### 2.3. IDA Impact Assessment Surveys (2022 and 2024–2025)

To evaluate the impact of MDA and assess the persistence of infection following cessation of treatment, cross-sectional IDA impact assessment surveys were conducted between April and May 2022 and again between December 2024 and January 2025. Both surveys targeted adults aged 20 years and older, in line with WHO guidance for evaluating the impact of the IDA regimen [14]. The country was divided into five evaluation units by grouping districts with similar ecological, epidemiological, and population characteristics. Within each evaluation unit, communities were selected at random using the Survey Sample Builder (SSB; NTD Support Center, Task Force for Global Health, Decatur, GA, USA; available at: https://www.cor-ntd.org/resources/transmission-assessment-survey-sample-builder; accessed on 22 July 2026) [21] and households were visited to recruit eligible participants. Circulating Filarial Antien (CFA) was detected through FTS (Alere Inc., Scarborough, ME, USA; now Abbott) testing of capillary blood obtained by finger prick. The FTS is a rapid diagnostic test widely used in LF surveillance programmes to detect circulating *W. bancrofti* antigen [22]. The FTS is the point-of-care assay recommended by WHO for the mapping and surveillance of bancroftian filariasis and has demonstrated high sensitivity for the detection of circulating filarial antigen compared with earlier immunochromatographic card tests [22]. In laboratory and field evaluation the FTS showed high sensitivity and specificity comparable to those of the reference ICT card test (over 99% agreement), while detecting a higher proportion of antigen-positive individuals in the field and offering greater result stability [22].

### 2.4. Detection of Microfilaremia

Individuals who tested positive for CFA during the surveys were invited to undergo nocturnal blood examination to detect microfilariae. Blood samples were collected at night, when *W. bancrofti* microfilariae are typically present in peripheral blood [14]. A calibrated thick blood smear was prepared from capillary blood, then examined under a microscope to detect and quantify microfilariae.

### 2.5. Morbidity Assessment

During the 2022 and 2024–2025 IDA impact assessment surveys, individuals were also screened for clinical symptoms consistent with LF, including lymphoedema of the limbs and hydrocoele in adult males. Information on identified cases was recorded to document the burden of morbidity and support MMDP activities within the national programme.

### 2.6. Data Sources

The data used in this study were obtained from multiple sources within the MoH of São Tomé and Príncipe. MDA coverage data for 2018, 2019 and 2020 were extracted from the Joint Reporting Forms submitted annually by the MoH to WHO as part of routine programme monitoring. Data from the IDA impact assessment surveys conducted in 2022 and between December 2024 and January 2025 were obtained from official survey reports prepared by the MoH with technical support from the WHO Country Office and the Expanded Special Project for Elimination of Neglected Tropical Diseases (ESPEN). Data on morbidity cases consistent with LF, including lymphoedema and hydrocoele, were ascertained through clinical screening of survey participants during the 2022 and 2024–2025 IDA impact assessment surveys rather than from routine service registers.

### 2.7. Data Management and Analysis

Survey data were recorded using standardized electronic data collection forms and entered into electronic databases for analysis. Data were collected electronically using the ESPEN Collect application. Antigen positivity was expressed as a percentage with exact numerators and denominators, and 95% confidence intervals (CIs) were calculated using the Wilson score method. All statistical analyses were performed in R version 4.3.0 (R Foundation for Statistical Computing, Vienna, Austria). Descriptive statistics were used to summarize the number of individuals tested, CFA prevalence, the presence of microfilaremia among antigen-positive individuals and the distribution of morbidity cases across health districts.

### 2.8. Ethics Statement

The surveys described in this study were conducted as part of national LF elimination programme activities implemented by the MoH of São Tomé and Príncipe in collaboration with WHO. These activities formed part of routine public health surveillance used to monitor programme performance and guide elimination strategies [14]. The impact assessment surveys were approved by the National Ethics Committee of São Tomé and Príncipe. Participation in the surveys was voluntary and verbal informed consent was obtained from all eligible participants before testing. Personal identifiers were not included in the analytical datasets used for this study.

## 3. Results

### 3.1. MDA Coverage (2018–2020)

Three consecutive rounds of MDA were implemented nationwide between 2018 and 2020. The first round in 2018 used the two-drug regimen of diethylcarbamazine and albendazole, while the second and third rounds in 2019 and 2020 used the IDA triple-drug regimen. Treatment was delivered in all seven health districts of São Tomé and Príncipe. Epidemiological coverage exceeded the WHO-recommended threshold of 65% in all health districts during each round of treatment [19]. National coverage reached 86.4% in 2018, 74.2% in 2019, and 80.0% in 2020. Coverage varied slightly across health districts but remained consistently high throughout the programme period (Table 1).

### 3.2. IDA Impact Assessment Survey (2022)

The first IDA impact assessment survey, conducted in 2022, examined 14,132 adults aged 20 years and older across five evaluation units. CFA was detected in 21 individuals, corresponding to an overall antigen positivity of 0.15% (21 of 14,132; 95% CI 0.10–0.23%). Antigen-positive individuals were identified in all evaluation units. The highest number of positive individuals was detected in the Caué–Lembá evaluation unit (eight cases), followed by Lobata–Mé-Zóchi (seven cases). Príncipe and Cantagalo reported three and two positive individuals respectively, while Água-Grande reported one positive individual. In all the evaluation units, the number of antigen-positive individuals remained below the critical cut-off values defined in the WHO decision framework [14] thresholds designed to ensure that infection prevalence is sufficiently low (generally around 1–2%) to prevent ongoing transmission—indicating that transmission is unlikely to be sustained (Table 2).

### 3.3. Follow-Up Impact Assessment Survey (2024–2025)

A follow-up survey conducted between December 2024 and January 2025 examined 14,653 adults aged 20 years and older across the same evaluation units. Seven individuals tested positive for CFA, corresponding to an overall antigen positivity of 0.05% (7 of 14,653; 95% CI 0.02–0.10%). Antigen-positive individuals were detected in four evaluation units: Príncipe (three cases), Cantagalo (two cases), Caué–Lembá (one case), and Lobata–Mé-Zóchi (one case). No positive individuals were detected in Água-Grande. All evaluation units remained below the WHO decision thresholds, confirming the findings from the initial IDA impact assessment survey and reinforcing the conclusion that LF transmission is unlikely to persist in the population (Table 2).

### 3.4. Microfilaremia Findings

All individuals who tested positive for CFA during the two surveys were invited to undergo nocturnal calibrated thick blood smear microscopy to detect microfilariae. A total of 28 antigen-positive individuals were examined, including 21 identified in 2022 and seven identified during the 2024–2025 survey. No microfilaria was detected in any of the blood smears examined, indicating the absence of detectable *W. bancrofti* microfilaremia among the antigen-positive individuals.

### 3.5. Morbidity Findings

During the 2022 and 2024–2025 IDA impact assessment surveys, clinical screening identified individuals with chronic symptoms of LF in all health districts of São Tomé and Príncipe. During the 2022 IDA impact assessment survey, 106 individuals with lymphoedema and 9 with hydrocoele were identified. The follow-up survey conducted in 2024–2025 identified 190 lymphoedema cases, while the number of hydrocoele cases remained unchanged at nine (Table 3). Lymphoedema cases were reported in all health districts in both datasets. In the 2022 survey, higher concentrations were found in Caué and Lembá, while the 2024–2025 survey showed more cases in Mé-Zóchi and Água-Grande. Hydrocoele cases were less common and more geographically concentrated, with a notable cluster in Príncipe during the 2022 survey and a more even distribution across health districts in 2024 (Table 3).

## 4. Discussion

The GPELF is built on two mutually reinforcing pillars: interrupting transmission through preventive chemotherapy and alleviating suffering via MMDP [7,23,24]. The findings of this study indicate that São Tomé and Príncipe has made substantial progress towards achieving the first pillar. Three consecutive rounds of nationwide MDA implemented between 2018 and 2020 consistently achieved high epidemiological coverage across all health districts, exceeding the thresholds required to interrupt transmission [17]. The subsequent introduction of the IDA regimen in 2019 and 2020 likely helped accelerate parasite elimination and reduce infection levels faster than the traditional two-drug regimens [12,13].

The results of the IDA impact assessment surveys conducted in 2022 and again between December 2024 and January 2025 showed extremely low levels of CFA across all evaluation units. In addition, nocturnal calibrated thick blood smear microscopy performed on antigen-positive individuals did not detect *W. bancrofti* microfilariae. The findings suggest that parasite transmission has declined to levels unlikely to sustain the parasite life cycle in the population. Similar declines in infection indicators have also been reported in other endemic settings following the introduction of the IDA regimen which aims to accelerate progress towards elimination [10,25,26,27]. Some limitations should be noted. Microfilaraemia was assessed using a single nocturnal calibrated thick blood smear; although appropriate for programmatic use, a single smear has limited sensitivity for low-density microfilaraemia and may fail to detect residual active infection in some antigen-positive individuals [14]. The absence of detectable microfilariae is therefore consistent with, but does not on its own confirm, interruption of transmission, and antigen positivity in a small number of adults indicates that low-level exposure cannot yet be fully excluded.

Several factors may have contributed to the progress observed in São Tomé and Príncipe. Experience from other elimination programmes has shown that sustained political commitment, strong programme leadership and high treatment coverage are key determinants of successful LF elimination efforts [28,29,30,31]. In São Tomé and Príncipe, the nationwide implementation of MDA, engagement of community health workers, and close collaboration between the MoH and international partners such as WHO facilitated effective programme implementation. The relatively small population size and insular geographic setting—similar to small island settings in the Western Pacific such as Niue, the Cook Islands and Samoa—may also have contributed to the rapid reduction in infection by limiting population movement and facilitating high nationwide treatment coverage [30,32,33].

São Tomé and Príncipe has concurrently pursued malaria elimination through integrated vector control, case management and targeted antimalarial mass drug administration [34,35,36,37]. Measures targeting *Anopheles mosquitoes*—larval source management, indoor residual spraying and long-lasting insecticidal nets—have achieved substantial coverage [38,39] and may have provided some incidental suppression of the vectors shared with lymphatic filariasis. However, these malaria vector control campaigns were largely implemented before the LF MDA rounds, and the present study was not designed to assess their contribution; any effect on LF transmission therefore cannot be quantified or attributed from these data, and MDA should be regarded as the principal driver of the reductions observed. The potential for operational synergy nonetheless supports continued coordination between the malaria and LF programmes.

While the programme has made significant progress towards interrupting transmission, the implementation of the second pillar of the elimination strategy, MMDP, remains limited. Individuals with chronic symptoms of LF, such as lymphoedema and hydrocoele, have been identified during the IDA impact assessment surveys. The lymphoedema and hydrocoele cases reported here were identified during the 2022 and 2024–2025 IDA impact assessment surveys and are considered to represent individuals who had already received MDA. Chronic lymphoedema and hydrocoele are largely irreversible sequelae of long-standing infection and are not expected to resolve following MDA; their continued identification therefore reflects accumulated historical morbidity rather than evidence of ongoing transmission. However, structured and accessible services for these individuals are not yet systematically integrated into the national health system. Addressing morbidity is a core requirement for validation of elimination, as the global strategy aims not only to interrupt transmission but also to alleviate suffering among affected individuals [40,41,42]. The national programme should therefore prioritize establishing and expanding morbidity management services across all health districts, including standardized care for lymphoedema and access to hydrocoele surgery. In parallel, documenting the availability, accessibility and quality of these services through facility assessments will be essential to demonstrate that MMDP components are functional and meet validation requirements.

Progress in this area has also been hampered by operational disruptions. Morbidity management activities initiated prior to the COVID-19 pandemic—particularly in Lobata and Lembá, where health personnel were trained and self-care materials were distributed—were disrupted and have not yet been fully reinstated nationwide. Strengthening efforts to resume case identification and expand service delivery will therefore be critical, both to improve patient care and to ensure that the programme can generate the evidence required for the national elimination dossier.

The timing of surveillance activities in São Tomé and Príncipe should also be aligned with WHO guidance, which recommends an interval of approximately four years between cessation of MDA and the final assessment of transmission [14]. Since MDA ended in 2020, the timing for this assessment was expected to be around 2024. Although there was a slight delay in implementing the IDA impact assessment surveys, with the latest survey being conducted between December 2024 and January 2025, the findings consistently indicate very low infection levels, supporting the conclusion that transmission has likely been interrupted.

Taken together, evidence from routine programme monitoring and the two IDA impact assessment surveys indicates that São Tomé and Príncipe is nearing the threshold required for validation of elimination of LF as a public health problem. The programme should now prioritize strengthening MMDP services while advancing the preparation of the national elimination dossier.

Looking ahead, several priorities will consolidate these gains. Post-validation surveillance should be established to detect any resurgence of transmission, including periodic antigen testing of young children born after cessation of MDA and, where feasible, entomological monitoring of the local *Anopheles* vectors, in line with WHO guidance [14]. In parallel, a minimum package of care for lymphoedema and hydrocoele should be embedded within routine primary health services and district hospitals, with trained staff, self-care support and reliable access to hydrocoele surgery, so that morbidity management is sustained beyond the vertical programme [4,6]. Completing and submitting the national elimination dossier, supported by facility-level documentation of these services, should now be the central objective of the programme.

## 5. Conclusions

The findings indicate that São Tomé and Príncipe has made substantial progress towards eliminating LF as a public health problem. High coverage levels achieved during three rounds of nationwide MDA between 2018 and 2020, followed by consistently low CFA levels and no detectable microfilaremia in post-MDA impact assessments conducted in 2022 and 2024–2025, suggest that transmission has likely been reduced to levels insufficient to sustain the parasite lifecycle. As the programme progresses towards validation, priority should now be given to strengthening MMDP services, including access to lymphoedema care and hydrocoele surgery, while also documenting service availability and quality. Advancing these efforts, together with preparing the national elimination dossier, will be essential to support formal validation by WHO.

## Figures and Tables

**Table 1:** MDA coverage by district, São Tomé and Príncipe, 2018–2020.

District	2018 (DA)			2019 (IDA)			2020 (IDA)		
	Target	Treated	Coverage (%)	Target	Treated	Coverage (%)	Target	Treated	Coverage (%)
Água-Grande	72,461	66,338	91.6	76,210	51,855	68	72,955	56,292	77.2
Mé-Zóchi	47,401	33,911	71.5	48,440	34,801	71.8	48,416	39,100	80.8
Lobata	20,508	18,635	90.9	21,015	16,204	77.1	21,075	16,059	76.2
Cantagalo	18,089	14,791	81.8	18,636	14,766	79.2	18,057	15,101	83.6
Caué	6717	6713	99.9	6376	6327	99.2	6702	6018	89.8
Lembá	15,177	14,192	93.5	16,099	12,357	76.8	14,929	12,288	82.3
Príncipe	7851	7931	101	7894	8217	104.1	7938	7261	91.5
Total	188,204	162,511	86.4	194,670	144,527	74.2	190,072	152,119	80

Note: DA = Diethylcarbamazine and Albendazole; IDA = Ivermectin, Diethylcarbamazine and Albendazole. Detailed coverage data provided in original manuscript.

**Table 2:** Results of IDA impact assessment surveys in adults aged 20 years and older, São Tomé and Príncipe, 2022 and 2024–2025.

Evaluation Unit	Health Districts Included	Survey 2022		Survey 2024–2025		Critical Cut-Off	Interpretation
		Adults Examined	Antigen-Positive	Adults Examined	Antigen-Positive		
Principe	Principe	1509	3	1589	3	9	Below threshold
Cantagalo	Cantagalo	3116	2	3581	2	18	Below threshold
Caué–Lembá	Caué, Lembá	3178	8	3282	1	18	Below threshold
Lobata–Mé-Zóchi	Lobata, Mé-Zóchi	3203	7	3006	1	18	Below threshold
Água-Grande	Água-Grande	3126	1	3195	0	18	Below threshold
Total	—	14,132	21	14,653	7	—	Transmission unlikely

**Table 3:** Distribution of lymphoedema and hydrocoele cases identified during the 2022 and 2024–2025 IDA impact assessment surveys, São Tomé and Príncipe.

District	Lymphoedema		Hydrocoele	
	2022	2024	2022	2024
Água-Grande	8	48	1	1
Mé-Zóchi	9	52	0	2
Lobata	10	28	1	1
Cantagalo	15	17	0	1
Caué	27	12	0	2
Lembá	26	21	1	1
Principe	11	12	6	1
Total	106	190	9	9

## Data Availability

All relevant data supporting the findings of this study are included within the manuscript.

## Supplementary Materials

The following supporting information can be downloaded at: https://www.mdpi.com/article/10.3390/tropicalmed11100262/s1. Figure S1: Pre-MDA endemicity status of lymphatic filariasis in São Tomé and Príncipe by health district (2015); Table S1: Results of the 2015 lymphatic filariasis confirmatory mapping by locality and health district, São Tomé and Príncipe (immunochromatographic antigen test, ICT).
