# Supplementary material for: São Tomé and Príncipe on the Verge of Eliminating Lymphatic Filariasis as a Public Health Problem: Evidence from IDA Impact Assessment Surveys
Source: Trop Med Infect Dis. 2026 Sep 23;11(10):262. doi: 10.3390/tropicalmed11100262 (PMC13709339; doi:10.3390/tropicalmed11100262)
Supplement: Supplementary file 1 [file tropicalmed-11-00262-s001.zip › tropicalmed-4433444-supplementary.pdf]

Supplementary Materials

São Tomé and Príncipe on the Verge of Eliminating Lymphatic Filariasis as a Public Health Problem: Evidence from IDA Impact Assessment Surveys

Supplementary Figure S1

Figure S1. Pre-MDA endemicity status of lymphatic filariasis in São Tomé and Príncipe by health district (2015).

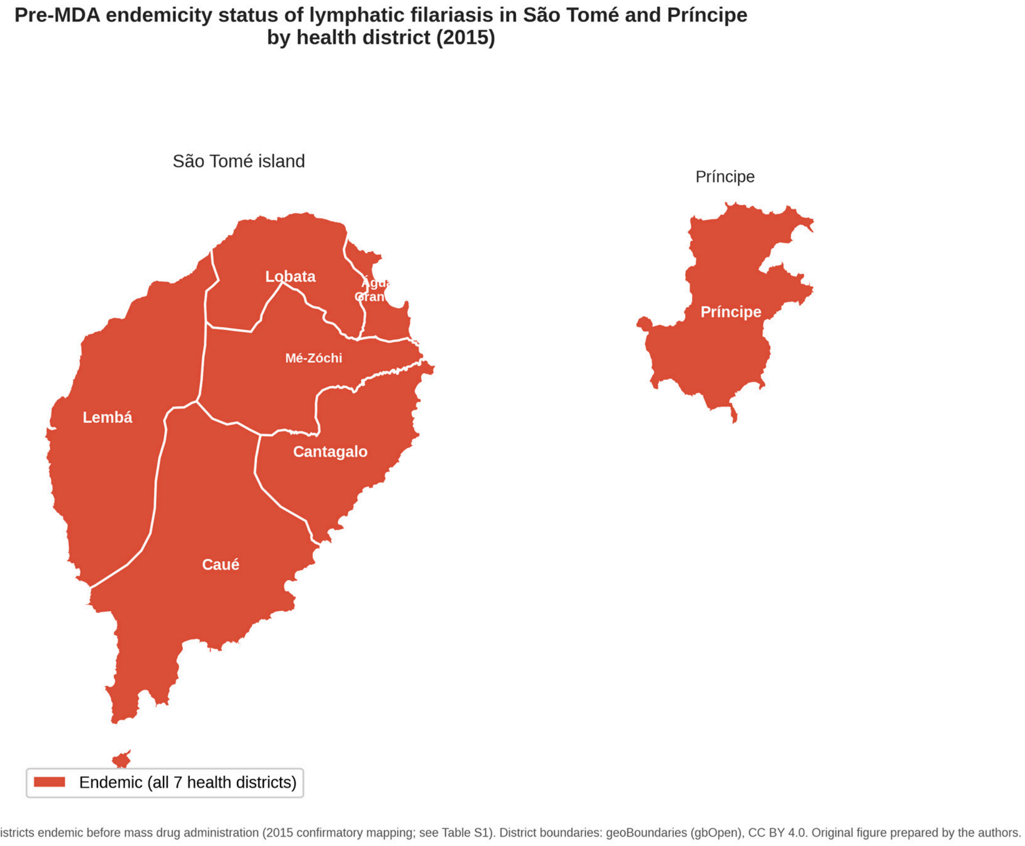

All seven health districts were classified as endemic before the start of mass drug administration, based on the 2015 confirmatory mapping (Table S1). São Tomé island comprises six health districts (Água Grande, Cantagalo, Caué, Lembá, Lobata and Mé-Zóchi) and Príncipe one (Pagué). District boundaries were derived from open administrative data (geoBoundaries, gbOpen; CC BY 4.0); the figure was prepared by the authors and contains no third-party copyrighted content.

Supplementary Table S1

Table S1. Results of the 2015 lymphatic filariasis confirmatory mapping by locality and health district, São Tomé and Príncipe (immunochromatographic antigen test, ICT).

Health district	Locality	Individuals tested (ICT)	ICT-positive	Antigen prevalence (%)	Endemicity
Água-Grande	Riboque Capital	100	1	1.00	Endemic
	Praia Gamboa	100	0	0.00	
Mé-Zóchi	Praia Melão	100	1	1.00	Endemic
	Cruzeiro	100	0	0.00	
Cantagalo	Ribeira Afonso	100	7	7.00	Endemic
	Riboque Santana	150	2	1.33	
Lobata	Agostinho Neto	92	0	0.00	Endemic
	Micoló	93	1	1.08	
Lembá	Diogo Vaz	78	0	0.00	Endemic
	Santa Catarina	100	7	7.00	
Caué	Porto Alegre	100	1	1.00	Endemic
	Angolares	100	1	1.00	
Príncipe	Praia Burra	100	1	1.00	Endemic
	Porto Real	100	1	1.00	
**Total (14 localities)**		**1,413**	**23**	—	**7/7 endemic**

ICT = immunochromatographic antigen test. Antigen prevalence is shown per surveyed locality (ICT-positive / tested). Following WHO recommendations, a district was classified as endemic when at least one surveyed community recorded at least one ICT-positive result; all seven health districts met this criterion and were included in nationwide mass drug administration.
